# Photo-derived transformation from modified chitosan*@*calcium carbonate nanohybrids to nanosponges

**DOI:** 10.1038/srep28782

**Published:** 2016-06-24

**Authors:** Jeong Hoon Byeon

**Affiliations:** 1 School of Mechanical Engineering, Yeungnam University, Gyeongsan 38541, Republic of Korea

## Abstract

Zwitterionic chitosan (ZC)*@*calcium carbonate (CC) nanoparticles were conveniently obtained and transformed to biocompatible nanosponges by continuous gas-phase photo-derived transformation in a single-pass configuration, and their potential use for biomedical applications was investigated. The mean diameter of the ZC*@*CC sponges was ~166 nm (~72 nm for CC and, ~171 nm for ZC), and the sponges had a mesoporous structure (i.e., an average pore diameter of ~13 nm). Measurements of the sponge cytotoxicity were performed and only a slight decrease was observed (>78% in cell viability) when compared with pure ZC (>80%). The ZC*@*CC sponges had a similar transfection ability to lipofectamine (~2.7 × 10^9^ RLU mg^−1^ protein) at a 50:1 ratio of sponge:DNA weight. Because of a porous structure, the sponges showed remarkably higher transfection efficiencies than pure ZC.

Recently, numerous approaches have been utilized to fabricate hybrid nanomaterials consisting of organic and inorganic components with desired sizes, shapes, and physicochemical and optical properties for their efficient use as functional materials and systems in various technological fields such as energy storage, biomedicine, micro- and nano-electronics^1^,^2^,^3^. Thus, finding versatile, tunable, and efficient strategies to prepare well-structured hybrid nanomaterials with appropriate functionalities is a very important issue facing materials technology, particularly nanoscience and nanoengineering^4^. In biomedical applications, the design of porous, biocompatible nanoplatforms that can form nanosponges for the efficient delivery of DNA, drugs, and other functional reagents is an area of much interest.

Calcium carbonate (CaCO_3_) (CC)-based inorganic-organic hybrid particles are biocompatible and have a unique structure that would be suitable to effectively protect DNA from damage during gene transfection into targeted cells^5^,^6^. CC is a common substance present in a range of natural products such as rocks, eggshells, and shells of marine organisms. Regarding industrial applications, CC particles are employed as a filler for various mixture compounds such as plastics, rubbers, and paints^7^. Chitosan is another abundant natural compound with a high biocompatibility, biodegradability, and antimicrobial activity, it is now intensively employed as a carrier or vehicle for various biomedical applications^8^. Moreover, a combination of CC and chitosan can have a strong potential for use in biomedical applications; however, to the best of our knowledge, the fabrication of CC-chitosan hybrid nanostructures for use in biomedical applications has not yet been reported.

Many formulations of inorganic-organic hybrid systems for biomedical applications are based on multistep wet chemistry and are introduced as the suspension of solid particles; these may only function as desired for a short period of time. Moreover, organic or polymeric components when incorporated with inorganic nanoparticles are normally unstable because of the gradual degradation by hydrolysis; therefore, hybrid nanomaterials in a suspension or colloidal form would not be recommendable^9^,^10^. In addition, nanomaterials in a colloidal form tend to aggregate during storage, changing the properties of materials and making them less suitable for bioapplications^11^,^12^. As a result, research is shifting toward preparation strategies that are simpler, more efficient, and more versatile to create stable hybrid nanomaterials for various biomedical applications. Gas-phase processing is a viable alternative that requires fewer preparation steps and enables a relatively better long-term storage of nanomaterials in the powder form.

Employing gas-phase processing enhances process continuity in production, implying that only simple mechanical collection of materials is required without producing much waste^13^. When using gas-phase processing in a single-pass configuration for the production of the inorganic component of the hybrid nanomaterials, it is essential to perform further treatments before the nanomaterials are suitable for use^2^. Recently, a combination of gas-phase strategies have introduced the possibility to fabricate porous microparticles (mostly silica based) for various therapeutic and diagnostic applications^14^,^15^. However, conventional gas-phase processing of nanomaterials is commonly performed under high-temperature conditions (at 500 °C and above), and thus, it would be only workable to fabricate hard or inorganic nanoparticles^16^. It is therefore necessary to use lower-temperature processing because temperatures above 300 °C can decompose most organic materials (i.e., biofunctional soft materials)^17^.

This study introduces a novel strategy to fabricate zwitterionic chitosan (ZC)@CC biocompatible nanosponges using continuous gas-phase processing in a serial reactor and explores its potential for gene transfection in biomedical systems with performing *in vitro* cytotoxicity testing. Unmodified chitosan is generally insoluble in water, and only sparingly soluble in organic liquids; thus, it is challenging to prepare chitosan-based hybrid materials^7^. Therefore, ZC, which consists of chitosan and succinic anhydride, is selected in this study because it has recently been highlighted as a modified chitosan that is soluble at neutral pH and is employed as a polymeric carrier^18^,^19^,^20^. CC nanoparticles were first generated by collison atomization before being injected into another collison atomizer filled with the ZC solution. Here, the CC nanoparticles incorporated with ZC precursors to form ZC@CC droplets. The droplets were then thermally cured in an electrically heated tube furnace at a 90 °C wall temperature under UV radiation to both extract the solvent from the droplets and form porous polymeric structures on the CC; this resulted in the formation of ZC@CC nanosponges. The sponges were collected to apply *in vitro* cytotoxicity and gene transfection in HeLa cells. Before *in vitro* measurements, the sampled sponges on a glass substrate were detached in an ultrasound bath to prevent unwanted agglomeration during storage. The results were compared with those of lipofectamine and pure ZC.

## Results and Discussion

**Figure 1:**
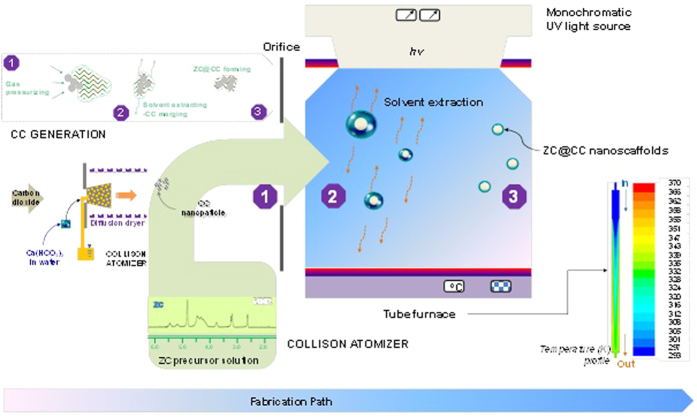
Schematic of continuous gas-phase processing to fabricate ZC@CC nanosponges using a series connection of two collison atomization systems.

Schematics of the continuous gas-phase processing used for these experiments are shown in Fig. 1. CC nanoparticles were first generated by an atomization system, which was achieved using a collison atomizer and a diffusion dryer. A pure carbon dioxide (99.999% purity) flow was passed throught a mass flow meter (3810DS, Kofloc, Japan) to control the flow rate to 3 L min^−1^. Moreover, 0.2 g of Ca(HCO_3_)_2_ dissolved water was atomized with carbon dioxide gas to form droplets and then passed through a diffusion dryer to extract the water. Proposed reactions to form CC nanoparticles are as follows:


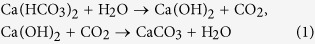


Dissolving supplied carbon dioxide into the solution may cause the transformation of intermediate Ca(OH)_2_ to CaCO_3_ during the reaction. The CC particle-laden flow was directly employed to further atomize the ZC precursor solution, which was prepared as detailed by Xu *et al*.^21^. The ZC@CC hybrid droplets then passed through a 254 nm-wavelength UV-irradiated (LOT-ORIEL, Germany) heated tubular reactor at 760 μW cm^−2^ intensity and 90 °C wall temperature with a 1.7 min residence time to apply phototreatment and simultaneous solvent extraction from the droplets. To promote the complete evaporation of the droplets, the required residence time in the tube furnace was estimated using equation 2. The time to saturate the gas with vapor from the evaporating droplets, *τ*, is given as


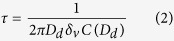


where *D*_d_ is the droplet diameter, *δ*_v_ is the vapor diffusivity, and *C*(*D*_d_) is the droplet number concentration. Figure 1 also shows the ^1^H NMR (Inova 300, Varian, US) spectrum of ZC. For pure chitosan (not shown), there were bands at chemical shifts around 2.6 ppm (-NHCOC**H**_2_C**H**_2_COOH), 4.0 ppm [H2 of N-acetyl glucosamine (GlcNAc), H3-H6 of GlcNAc, and glucosamine (GlcN)], and 4.8 ppm (H1 of GlcN). On the other hand, ZC (An/Am = 0.5, molar ratio of succinic anhydride to chitosan amine) had additional bands at chemical shifts of 3.15 ppm (H2 of GlcN) and 4.3 ppm (-N**H**COCH_2_CH_2_COOH)^22^.

**Figure 2:**
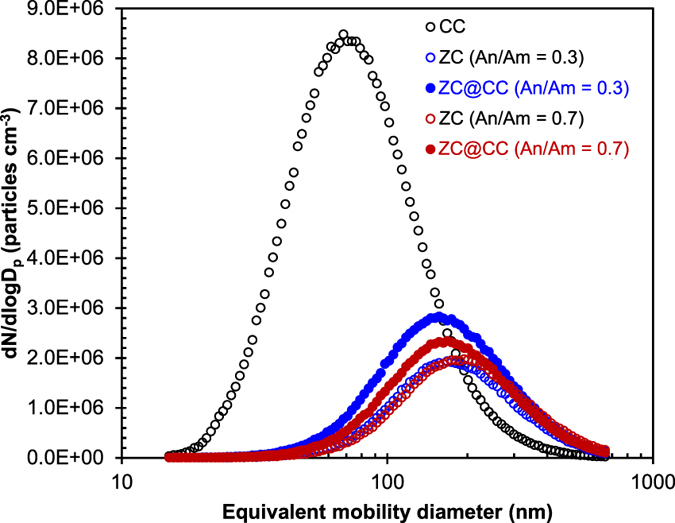
Size distributions of ZC@CC particles in the gas-phase in comparison with pure CC and ZC particles.

Figure 2 summarizes size distribution measurements of the ZC@CC particles. The distribution was analyzed using a scanning mobility particle sizer (3936, TSI, US) to verify mean diameter, standard deviation, and number concentration of the ZC@CC particles (An/Am = 0.3), which were 169.2 nm, 1.71, and 1.74 × 10^6^ cm^−3^, respectively. Analogous data for CC was 74.7 nm, 1.73, and 4.94 × 10^6^ cm^−3^, respectively, and for ZC (An/Am = 0.3), it was 176.5 nm, 1.67, and 1.06 × 10^6^ cm^−3^, respectively. The data for the ZC@CC particles was closer to that of pure ZC particles rather than pure CC particles. There was no additional peak, and this suggests that CC particles were well merged with ZC, to transform to ZC@CC particles. The other data for the “An/Am = 0.7” cases is described in Supplementary Table S1. The mass fractions of CC and ZC in the ZC@CC particles were measured using a piezobalance particle monitor (3522, Kanomax, Japan) to be 0.71 (0.68) and 0.29 (0.32) for An/Am = 0.3 (An/Am = 0.7), respectively.

**Figure 3:**
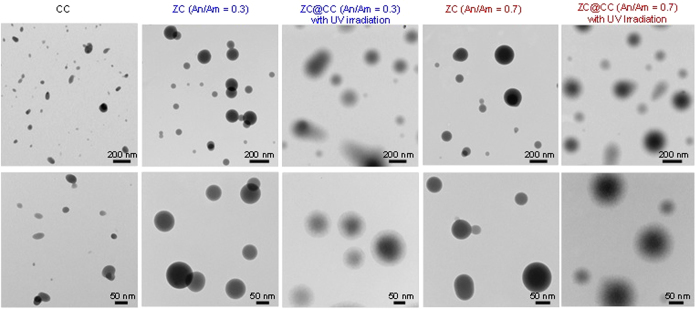
Low- and high-magnification TEM images of ZC@CC sponges in comparison with pure CC and ZC particles.

**Figure 4:**
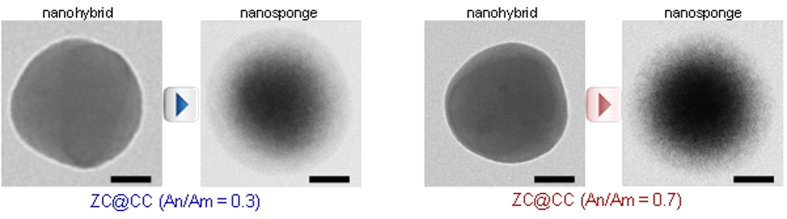
High-magnification TEM images (scale bar, 50 nm) without and with UV irradiation in the heated tubular reactor. The morphology of ZC@CC nanohybrids were changed to porous structures, “nanosponges”, through photo-derived reaction of ZC on CC particles.

Transmission electron microscope (TEM, CM-100, FEI/Philips, US) images (Fig. 3) indicate that most CC particles have an elliptical shape, whereas ZC particles exhibited a spherical shape and had a smooth surface. Nevertheless, both particles were well separated. For ZC, particles had a gradation (dark core-dense solid, bright shell-light solid), which were obtained by this method because of the given drying rate and can be explained with the Peclet number, *Pe*, which is a dimensionless number that represents relative time-scales for diffusion (*D*_d_^2^/4*δ*_v_) and convective drying (*τ*_d_).


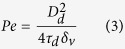


the *Pe* value of the current condition was significantly smaller than 1; this confirmed that the migration of solutes at the interface toward the core region of the droplets was sufficient to stay with the convective drying, thereby inducing the formation of dense solid particles. When CC particles were injected into the other atomizer (refer Fig. 1), they were encapsulated by ZC droplets because of the gas pressurizing system. In the case of ZC@CC sponges, the high-magnification TEM image shows the gray network shell around CC core particles, implying the presence of a ZC moiety that completely covered CC particles. The efficiencies of ZC in the encapsulation of CC particles for An/Am values of 0.3 and 0.7 were 97.9% and 98.7%, respectively. The mean diameters of pure CC and ZC were 72 ± 5.1 and 171 ± 9.3 nm, respectively. The analogous data for the ZC@CC sponges was 166 ± 9.1 nm, which is consistent with the data described in Fig. 2 and Supplementary Table S1. Figure 4 shows high magnification TEM images without and with UV irradiation in the heated tubular reactor, which supports morphological changes of ZC@CC nanohybrids through photo-derived transformation of ZC components on CC particles in the gas-phase.

**Figure 5:**
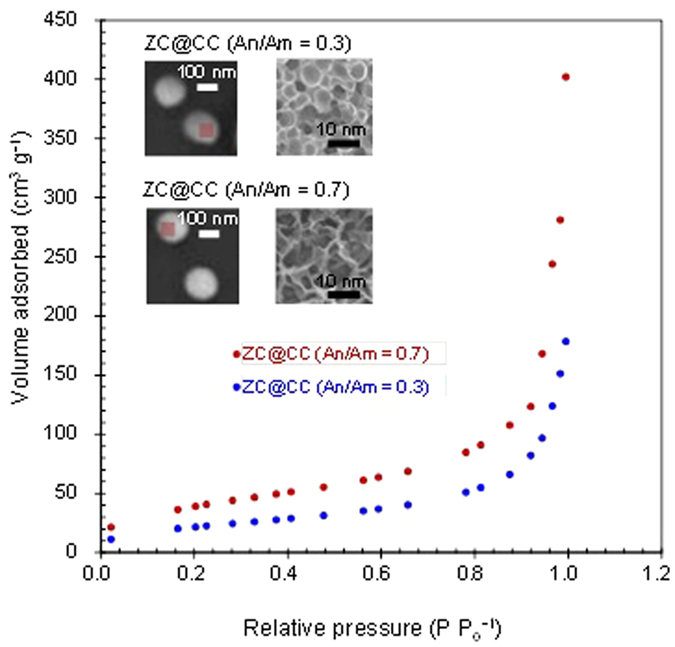
Adsorption isotherms of ZC@CC sponges and corresponding SEM images.

Figure 5 shows the textural properties of ZC@CC sponges at 77.4 K. The adsorbed volume first increased at 0–0.05 at relative pressure (P P_0_^−1^); this may be related to rapid adsorption in the micropores of the sponges. The adsorbed volume then slowly increased at 0.05–0.85 at P P_0_^−1^ owing to capillary suction in the mesopores of the sponges. The volume drastically increased further at 0.9–0.99 owing to strong capillary condensation^23^. Supplementary Table S2 summarizes textural properties such as surface area, pore volume, and diameter. The insets of Fig. 5 show scanning electron microscopy (SEM, NOVA nanoSEM, FEI, US) images of the ZC@CC sponges, which clearly show a porous structure on CC particles; on the other hand, the other CC-chitosan combinations did not show any porous structure on CC particles (Supplementary Fig. S1). Two possible explanations for this phenomenon are 1) the succinyl group may be more sensitive to UV irradiation than other groups and 2) ZC is more soluble at neutral pH and thus may induce a preference for the formation of a porous structure. A greater porosity in the case of An/Am = 0.7 than in the case of An/Am = 0.3 further demonstrates the above explanations.

**Figure 6:**
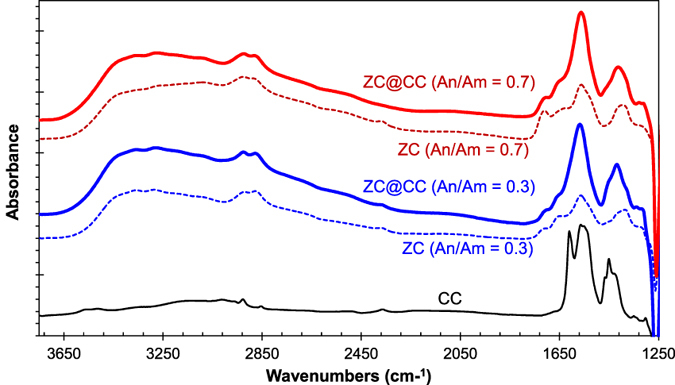
FTIR spectra of ZC@CC sponges in comparison with pure CC and ZC particles.

In Fig. 6, the FTIR spectra of ZC exhibits a prominent band at 1540 cm^−1^, which can be indexed by the bending vibration of N-H amides (amide II) due to the existence of succinyl groups (*N*-succinylation)^24^. Moreover, the spectra exhibit typical absorption bands at 3360, 2920, 2880, 1680, and 1360 cm^−1^, which represent the -OH, -CH_2_, -CH_3_, amide I, and amide III groups of pure chitosan, respectively^25^. A peak at 1440 cm^−1^ is attributed to the carbonate group, indicating that the crystalline of CC is calcite^6^,^26^. After ZC incorporated CC particles, the spectra of amide II and III groups intensified remarkably while the band for CC disappeared. This implies that nearly all CC particles were quantitatively covered by ZC. The shifting of the peaks at 1360 and 1540 cm^−1^ to higher wavenumbers (1380 and 1550 cm^−1^) indicates associative effect between ZC and CC. The weakened peak at 1680 cm^−1^ also suggests coordination between ZC and CC^27^. In addition, the zeta potentials of particle/pDNA complexes are described in Supplementary Table S3, and there was no significant difference between pure ZC particles and ZC@CC sponges; this implies that the merging did not affect the surface chemistry.

**Figure 7:**
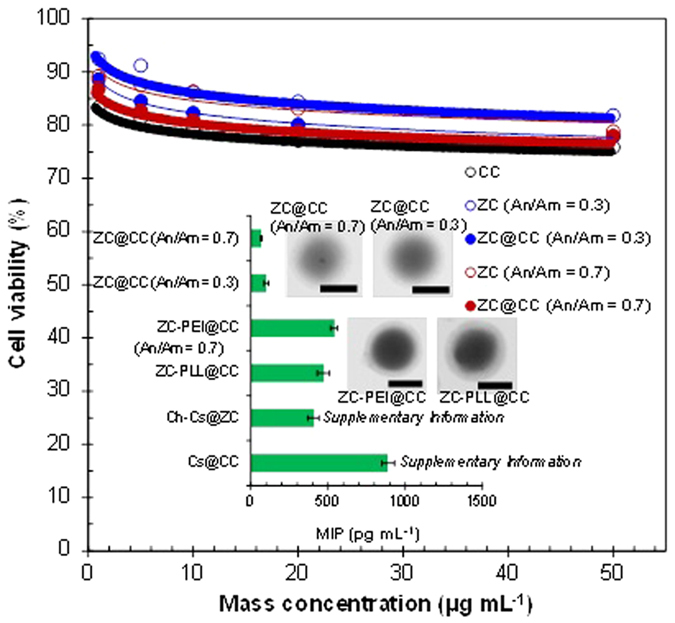
*In vitro* cytotoxicities of ZC@CC sponges in comparison with pure CC and ZC particles. Insets show MIP production from LPS-challenged macrophages by adding ZC@CC sponges or other controls [ZC-PEI (adding 2 × 10^−6^ mol dm^−3^)@, ZC-PLL (adding 2 × 10^−6^ mol dm^−3^)@, Ch-Cs@, and Cs@CC particles]. Insets also show representative TEM images (scale bar, 100 nm) of the ZC@CC (An/Am = 0.7), ZC@CC (An/Am = 0.3), ZC-PEI@CC (An/Am = 0.7), and ZC-PLL@CC (An/Am = 0.7).

The dynamic light scattering measurements of the nanosponges were firstly performed. The directly gas-phase sampled nanosponges on a glass plate were applied just before *in vitro* measurements. The results showed that the deviation of hydrodynamic diameter is no larger than 3.4% for all the tested nanosponges, and there are no significant differences between the storage days (1–14 days). This implies that the nanosponges have stability that warrants further investigation. The cytotoxicity of the sponge/pDNA complexes was determined in HeLa cells using MTS assays at different concentrations, such as 1, 5, 10, 20, and 50 μg mL^−1^ and compared to those from pure CC and ZC (Fig. 7). To prepare the particle solution, sample particles were first detached from the hydrophobic substrate (i.e., glass plate) by dipping the substrate into water for 1 min under ultrasonification. The measurement results reveal that the cell viability was >78% for the ZC@CC sponges, whereas the analogous data of pure CC and ZC particles were >75% and >80%, respectively. The lowest viability was for pure CC, which indicates that it may induce oxidative damage to HeLa cells, as reported in a previous study^28^, and this may affect the cytotoxicity of the ZC@CC sponges. Nevertheless, the results imply that the fabricated ZC@CC sponges had a biocompatibility that may be non-cytotoxic, and in principle, there was no remarkable difference of cell viability between the ZC@CC and pure ZC. Furthermore, another scenario for parenteral applications was considered, where ZC@CC sponges are administered to tissues that attract activated macrophages. In order to suppress inflammatory responses, nanoparticle interaction with biological system has recently been studied for nanoparticulate delivery systems^29^. The results (inset of Fig. 7) show that ZC@CC sponges could more significantly suppress the macrophage inflammatory protein (MIP) production from lipopolysaccharide (LPS)-challenged macrophages than those from unmodified chitosan (Cs) and cholesterol modified chitosan (Ch-Cs) shown in Supplementary Fig. S1. This may be due to binding between the ZC surfaces and the cell surface receptors and/or the LPS^30^ that regulate MIP production. Even smaller MIP productions of ZC@CC sponges than those from ZC-polyethyleneimine (PEI) and –poly-l-lysine (PLL) indicate that the tendency may be due to larger amine contents, and this also supports the higher MIP values from Cs@ and Ch-Cs@CC particles.

**Figure 8:**
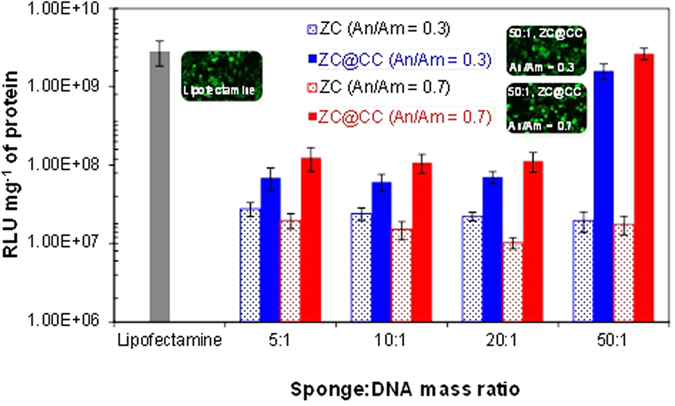
*In vitro* gene transfection efficiencies of ZC@CC sponges in comparison with lipofectamine (as positive control) and pure ZC particles. Transfection fluorescence imaging (insets) of lipofectamine and ZC@CC (50:1) samples in HeLa cells for 24 h.

To confirm the biomedical potential of the ZC@CC sponges, transfection efficiencies were further measured in HeLa cells using pDNA that contained luciferase and green fluorescent protein (GFP) gene. The transfection efficiencies of ZC@CC sponges for sponge:DNA weight ratios <25:1 in the HeLa cell line were remarkably lower than those when lipofectamine was used as the positive control (Fig. 8). The efficiency for the 50:1 case reached a similar degree as that of lipofectamine. Figure 8 also shows GFP-derived fluorescence images of HeLa cells for lipofectamine or ZC@CC (50:1) complexes, which further confirmed the transfection and similarities between the lipofectamine and ZC@CC complexes. The higher efficiencies of the ZC@CC sponges compared with pure ZC can be ascribed to the high capacity to carry GFP genes due to a higher porosity because there was no significant difference in the zeta potential between pure ZC particles and ZC@CC sponges. A greater transfection ability in An/Am = 0.7 compared to An/Am = 0.3 may also have originated from a greater porosity, although the transfection ability in An/Am = 0.3 was greater than that in An/Am = 0.7 for pure ZC because of a difference in the zeta potentials. According to negative charges of the particles at pH 7.4 including large sizes (~170 nm), modulations of size and porosity are underway for efficient gene/drug delivery applications without increases in sponge:DNA ratios as well as toxicological/inflammatory responses. An electrophoretic mobility shift experiment (Supplementary Fig. S2) was performed to confirm the formation of the nanosponge-gene complexes regarding the sponge:DNA ratios employed in the gene transfection tests. The 50:1 ratio was more effective to bind genes than those in other ratios. This was consistent with the gene transfection tests (Fig. 8), since capillary suction^31^ of the sponges to carry genes at the smaller ratios may not be sufficient, and showed significant gene liberation from the combinations.

For the first time, continuous gas-phase processing under UV irradiation was performed to fabricate biocompatible ZC@CC nanosponges. These sponges from the photo-derived transformation were employed in *in vitro* measurements of biocompatibility and transfection efficiency. The mean diameter of the ZC@CC sponges was ~166 nm (~72 nm for CC and, ~171 nm for ZC), and the sponges had a mesoporous structure (i.e., an average pore diameter of ~13 nm). *In vitro* measurements revealed that the fabricated sponges had slightly higher cytotoxicity (~78% in the minimum cell viability; ~75% for pure CC) than pure ZC (~80% in the minimum cell viability). The sponges reached a similar transfection ability as lipofectamine (~2.7 × 10^9^ RLU mg^−1^, as positive control) at a 50:1 sponge:DNA weight ratio, this was probably because of the porous structure. The fabricated sponges showed remarkable enhancement in gene transfection efficiency in comparison with pure ZC. These results will establish continuous single-pass processing as an efficient, environmentally friendly, and versatile means to design, fabricate, and modify this methodology, which is generalizable to a wide range of biofunctional nanomaterials.

## Additional Information

**How to cite this article**: Byeon, J. H. Photo-derived transformation from modified chitosan@calcium carbonate nanohybrids to nanosponges. *Sci. Rep*. **6**, 28782; doi: 10.1038/srep28782 (2016).

## Supplementary Material

Supplementary Information
