# Supplementary material for: Photo-derived transformation from modified chitosan@calcium carbonate nanohybrids to nanosponges
Source: Sci Rep. 2016 Jun 24;6:28782. doi: 10.1038/srep28782 (PMC4919630; doi:10.1038/srep28782)
Supplement: Supplementary Information [file srep28782-s1.pdf]

Supplementary Information for Publication

Photo-derived transformation from modified chitosan@calcium carbonate nanohybrids to nanosponges

Jeong Hoon Byeon[*]

School of Mechanical Engineering, Yeungnam University, Geongsan 38541, Republic of Korea

[*]E-mail: postjb@yu.ac.kr

- *Instrumentation*

The size distributions of the fabricated particles were measured using a scanning mobility particle sizer (SMPS), consisting of a differential mobility analyzer (3081, TSI, US), electrostatic classifier (3080, TSI, US), condensation particle counter (3776, TSI, US), and a soft X-ray charger (4530, HCT, Korea). The SMPS system, which was used to measure the mobility equivalent diameter, was operated at a sample flow of 0.3 L min^{-1}, a sheath flow of 3.0 L min^{-1}, and a scan time of 135 sec (measurement range: 15.1-661.2 nm). The mass (m) of the fabricated particles was measured using a microbalance (DV215CD, Ohaus, Switzerland) and also confirmed *via* the following equation:

$$m = Q \cdot t_s \int_0^{\infty} \eta(D_p) C_m(D_p) dD_p \;) \qquad \text{(S1)}$$

where Q is the flow rate of carbon dioxide gas, t_s is the sampling time, $\eta(D_p)$ is the fractional collection efficiency, and $C_m(D_p)$ is the mass concentration of particles.

Transmission electron microscope (TEM, CM-100, FEI/Philips, US) images were obtained at an accelerating voltage range of 46-180 kV. Specimens were prepared for examination in the TEM by direct electrostatic gas-phase sampling at a sampling flow of 1.0 L min^{-1} and an operating voltage of 5 kV using a nano particle collector (NPC-10, HCT, Korea).

Scanning electron microscope (SEM, NOVA nanoSEM, FEI, US) images for the CC-ZC particles were obtained at an accelerating voltage of 15 kV. The nitrogen adsorption isotherms of the ZC@CC sponges were measured using a porosimeter (ASAP 2010, Micromeritics Ins. Corp., US) at 77.4 K at a relative pressure ranging from 10^{-6} to 1.

For Fourier transform infrared (FTIR) spectroscopy analysis, samples were prepared using polytetrafluoroethylene (PTFE) media substrate (0.2 μm pore size, 47 mm diameter, 11807-47-N, Sartorius, Germany) by physical filtration (*i.e.* mechanical filtration mainly by diffusion, of particles on the surfaces of the substrate), and the spectra were recorded on a Nicolet 6700 FTIR spectrometer (Thermo Electron, US). The spectra were taken for samples in the range of

4000-400 cm^{-1} in absorbance mode.

The zeta potential of sponge/plasmid DNA (pDNA) complexes was determined using a zeta potential analyzer (Nano ZS-90, Malvern Instruments, UK). The particles were mixed with pDNA, and incubated at room temperature for 30 min. The complexes were then diluted with double de-ionized water to an appropriate concentration. Measurements of the zeta potential were carried out at 25°C and calculated using the manufacturer's supplied software.

- *Agarose Gel Retardation Assay*

The gene condensation ability of the nanosponges under different weight ratios were analyzed by 1% agarose gel electrophoresis using tris-acetate-ethylenediaminetetraacetic acid buffer (242 g Tris, 57.1 mL glacial acetic acid, and 0.5 mM ethylenediaminetetraacetic acid, pH 8.0) containing 0.5 µg mL^{-1} ethidium bromide. Complexes containing nanosponges and genes with different weight ratios were prepared by mixing, voltexing, and incubating them at room temperature for 30 min. Approximately 100 ng of each complex was loaded on agarose gels. A gel loading dye blue (New England BioLabs, USA) was added to each well and agarose gel electrophoresis was carried out at a constant voltage of 80 V for 50 min. The gene bands of the resultant gels were then visualized under a ultraviolet transilluminator at a wavelength of 365 nm.

- ***In Vitro* Cytotoxicity and Transfection**

The cytotoxicity of the ZC@CC nanosponges was evaluated using HeLa cells by the MTS, 3-(4,5-dimethyl-thiazol-2-yl)-5-(3-carboxymethoxyphenyl)-2-(4-sulfophenyl)2H-tetrazolium, assay. The cells were cultured in 200 mL of Dulbecco's modified eagle medium (DMEM Carlsbad, USA) supplemented with 10% fetal bovine serum (FBS) at 37℃, 5% CO_2, and 95% relative humidity. The cells were seeded in a 96-well microtiter plate (Nunc, Germany) at densities of 1×10^5 cells well^{-1}. After 24 h, the culture media were replaced with serum-supplemented culture media containing the nanosponges (1mg mL^{-1}), and the cells were incubated for 24 h. Then, 30 µL of the MTS reagent was added to each well. The cells were incubated for an additional 2 h. The absorbance was then measured using a microplate reader (Spectra Plus, TECAN, Switzerland) at a wavelength of 490 nm. The cell viability (%) was compared with that of the untreated control cell in media without nanosponges and calculated with $[A]_{test}:[A]_{control} \times 100\%$, where $[A]_{test}$ is the absorbance of the wells with nanosponges and $[A]_{control}$ is the absorbance of the control wells.

The ability of ZC@CC nanosponges to transfect HeLa cells using pDNA that contain the firefly luciferase and GFP gene. The cells were seeded at a density of 1×10^6 cells well^{-1} in 24-well plate in RMPI 1640 medium (Gifco, USA) containing 10% FBS, and grown to reach 80% confluence prior to transfection. Before transfection, the medium was exchanged with fresh medium with 10% FBS. The cells were treated with the nanosponge solution containing 2 μg of pDNA for 4 h at 37°C and the final volume was adjusted to 500 μL by medium. After exchanging with a fresh medium with 10% FBS, cells were further incubated for 48 h. Then the growth medium was removed, and the cells were shaken for 30 min at room temperature in 200 μL of Reporter Lysis Buffer (Promega, USA). The lysates were transferred into tubes and centrifuged at 13,000 rpm for 5 min. Luciferase activity was measured with a luminometer (TD-20/20, Promega, USA). The total protein was determined by BCA protein

assay kit (ThermoFisher Scientific, USA). The final luciferase activity was expressed as relative luminescence units (RLU) mg^{-1} protein. Inverted fluorescent microscope (Nikon Eclipse TE2000-S, Japan) was used to observe the GFP expression of the nanosponges in HeLa cells.

All experiments were performed in triplicate, and the results were reported as means and standard deviations. Statistical analyses were performed using Student's *t*-test. The differences were considered significant for $p < 0.05$.

- ***Macrophage Inflammatory Protein (MIP) Production***

Peritoneal macrophages were seeded in 24-well plates at a density of 10^5 cells per well in 1 mL of medium. After overnight incubation, 0.1 mL of the Janus particle solution was injected to each well to set the particle concentraion in medium to 2 mg mL^{-1}. For comparison purposes, 0.1 mL of polyethyleneimine (PEI, 765090, Sigma-Aldrich, USA), poly-l-lysine (PLL, P4707, Sigma-Aldrich, USA), or polyethylene glycol (PEG, 81188, Sigma-Aldrich, USA) was injected in lieu of the ZC precursor solutions. After 24 h incubation, the culture media were centrifuged at 2000 rpm for 10 min to separate supernatants. Macrophages were challenged by adding lipopolysaccharide (LPS) to the media in the final concentration of 1 μg mL^{-1} shortly before the comparisons. Enzyme-linked immunosorbent assay (ELISA) was performed to determine the MIP levels using MIP-2 ELISA kit (R&D Systems, USA). The supernatants collected from LPS-challenged macrophages was always diluted 10 times prior to the analysis. The differences were considered significant for $p < 0.01$.

- ***TEM images for the other chitosan@CC nanohybrids***

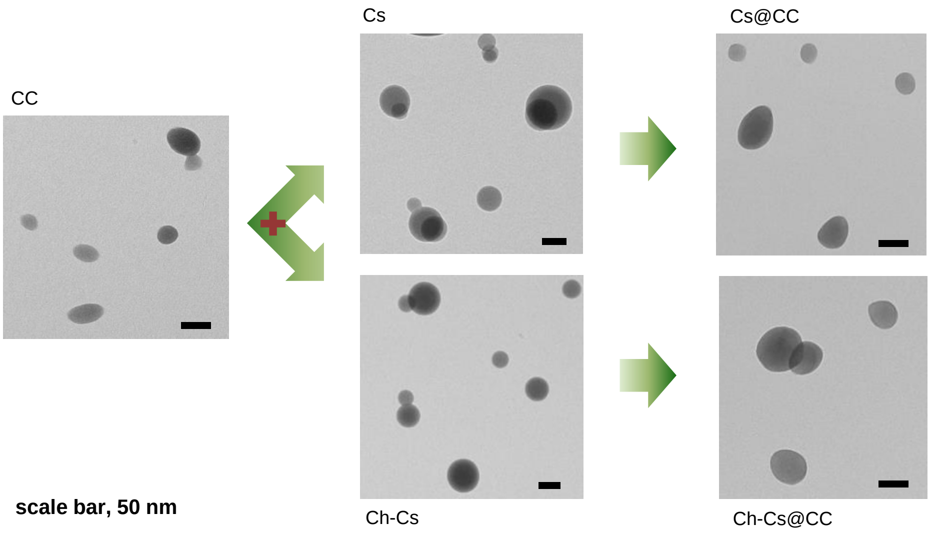

Figure S1. TEM images for the other chitosan@CC nanohybrids.

The TEM images (**Fig. S1**) indicate that the morphology of the CC particles is an elliptical shape, while pure chitosan (Cs) and cholesterol-chitosan (Ch-Cs) particles exhibit a similar spherical shape with a smooth surface and are separate from each other. When the CC particles passed through the orifice of the atomizer, the CC particles were capsulated by Cs or Ch-Cs particle due to the gas pressurizing system. The TEM images show the gray outer shell around CC nanoparticles, implying the presence of a Cs or Ch-Cs moiety that completely covers the CC particles. However, there was no porous chitosan network on the CC particles, dissimilar to the ZC@CC configuration, although they were fabricated by the same method used to fabricate the ZC@CC. This difference may have originated from the differences in the UV sensitivity and solubility in water among the configurations.

- ***Agarose gel retardation assay***

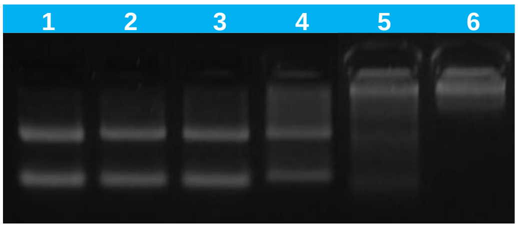

Figure S2. Gel retardation assay of nanosponge/pDNA combinations for An/Am = 0.7. Lane 1 is pDNA, and lanes 2-6 are nanosponge/pDNA combinations with the mass ratios of 5:1, 10:1, 20:1, and 50:1.

- ***Supplementary Tables***

Table S1 Summary of aerosol size distributions of ZC@CC sponges in comparison to pure CC and ZC particles

Case	GMD (nm)	GSD (-)	TNC (particles cm^{-3})
CC	74.7	1.73	4.97×10^{6}
ZC (An/Am = 0.3)	176.5	1.67	1.06×10^{6}
ZC@CC (An/Am = 0.3)	169.2	1.71	1.74×10^{6}
ZC (An/Am = 0.7)	189.7	1.69	1.12×10^{6}
ZC@CC (An/Am = 0.7)	169.6	1.70	1.33×10^{6}

Table S2 Textural properties of ZC@CC sponges

Case	Total		Microporous		Mesoporous		Average Pore Diameter
	SA, $m^2\ g^{-1}$	PV, $cm^3\ g^{-1}$	SA, $m^2\ g^{-1}$	PV, $cm^3\ g^{-1}$	SA, $m^2\ g^{-1}$	PV, $cm^3\ g^{-1}$	nm
ZC@CC (An/Am = 0.3)	123.58	0.346	28.43	0.025	95.15	0.321	12.16
ZC@CC (An/Am = 0.7)	182.73	0.529	38.57	0.088	144.16	0.441	14.71

Table S3 Zeta potential of ZC@CC-pDNA combinations in comparison to pure CC and ZC particles

Combinations	Zeta potential (mV)
CC	-1.89 ± 0.29
ZC (An/Am = 0.3)	-15.6 ± 2.85
ZC@CC (An/Am = 0.3)	-14.3 ± 2.22
ZC (An/Am = 0.7)	-39.1 ± 5.58
ZC@CC (An/Am = 0.7)	-39.5 ± 4.21
